# Racism, African American Women, and Their Sexual and Reproductive Health: A Review of Historical and Contemporary Evidence and Implications for Health Equity

**DOI:** 10.1089/heq.2017.0045

**Published:** 2018-09-24

**Authors:** Cynthia Prather, Taleria R. Fuller, William L. Jeffries, Khiya J. Marshall, A. Vyann Howell, Angela Belyue-Umole, Winifred King

**Affiliations:** ^1^ Division of HIV/AIDS Prevention, National Center for HIV, Hepatitis, STD and TB Prevention, Centers for Disease Control and Prevention, Atlanta, Georgia.; ^2^ Division of Reproductive Health, National Center for Chronic Disease Prevention and Health Promotion, Centers for Disease Control and Prevention, Atlanta, Georgia.; ^3^ Division of Violence Prevention, National Center for Injury Prevention and Control, Centers for Disease Control and Prevention, Atlanta, Georgia.; ^4^ Division of Global HIV and TB, Center for Global Health, Centers for Disease Control and Prevention, Atlanta, Georgia.

**Keywords:** African American women, racism, sexual and reproductive health

## Abstract

**Background:** The sexual and reproductive health of African American women has been compromised due to multiple experiences of racism, including discriminatory healthcare practices from slavery through the post-Civil Rights era. However, studies rarely consider how the historical underpinnings of racism negatively influence the present-day health outcomes of African American women. Although some improvements to ensure equitable healthcare have been made, these historical influences provide an unexplored context for illuminating present-day epidemiology of sexual and reproductive health disparities among African American women.

**Methods:** To account for the unique healthcare experiences influenced by racism, including healthcare provision, we searched online databases for peer-reviewed sources and books published in English only. We explored the link between historical and current experiences of racism and sexual and reproductive health outcomes.

**Results:** The legacy of medical experimentation and inadequate healthcare coupled with social determinants has exacerbated African American women's complex relationship with healthcare systems. The social determinants of health associated with institutionalized and interpersonal racism, including poverty, unemployment, and residential segregation, may make African American women more vulnerable to disparate sexual and reproductive health outcomes.

**Conclusions:** The development of innovative models and strategies to improve the health of African American women may be informed by an understanding of the historical and enduring legacy of racism in the United States. Addressing sexual and reproductive health through a historical lens and ensuring the implementation of culturally appropriate programs, research, and treatment efforts will likely move public health toward achieving health equity. Furthermore, it is necessary to develop interventions that address the intersection of the social determinants of health that contribute to sexual and reproductive health inequities.

*What happened on that auction block centuries ago is still unfinished business for African American women today.*
—*Dr. Gail E.* *Wyatt^1^*

## Introduction

Racism in the United States is pervasive and is a major contributor to sexual and reproductive health disparities of African American women. The historical narrative about racial inferiority has exacerbated discriminatory healthcare practices, in turn negatively affecting the quality and types of healthcare provided to African American women.^2–6^ According to the Centers for Disease Control and Prevention (CDC), African American women experience a high burden of maternal mortality, infant mortality, and sexually transmitted infections (STI), including HIV.^4–9^ Furthermore, racism is a fundamental determinant of health status because it contributes to social inequalities (e.g., poverty) that shape health behaviors, access to healthcare, and interactions with medical professionals.^3,10,11^

Although legalized slavery, the most salient manifestation of race-based mistreatment for African Americans, ended in 1865, racism persists in institutions (e.g., criminal justice system), and attitudes that marginalize African American women.^4,12,13^ For this reason, a historical analysis might shed light on how current sexual and reproductive health outcomes for African American women are shaped by racism and inform public health interventions to improve outcomes and promote health equity.

## Methods

First, we highlight a combination of significant historical events throughout four key eras that play a role in current health outcomes, including slavery, Black Codes/Jim Crow, Civil Rights, and post-Civil Rights (present day). The authors posit that a combination of these race-based events across eras impacts the current reproductive and sexual health status of African American women. We searched online databases (e.g., PubMed) for peer-reviewed sources and books published in English only. To account for the unique healthcare experiences influenced by racism, including healthcare provision and research, our search was limited to the United States only. Second, we describe contemporary sexual and reproductive health outcomes. Third, we explore the link between these historical experiences and current sexual and reproductive health outcomes. Finally, we discuss the potential benefit for public health interventions that acknowledge the historical and current health status and healthcare experiences of African American women, and interventions that promote health equity.

We argue that a careful examination of historical factors is essential to effectively address the current healthcare needs of African American women especially as they relate to chronic stress and impacts on health outcomes across a variety of conditions potentially rooted in racism, including STI (e.g., HIV) and pregnancy-related morbidity and mortality. If past influences that have potentially shaped current outcomes are not taken into consideration, then public health efforts may neglect the impact of larger, contextual factors that affect health and contribute to inequities. Given the nature of this article, our review was considered exempt by the institutional review board and not required.

## Results

### Key historical considerations: slavery to present

**FIG. 1.**
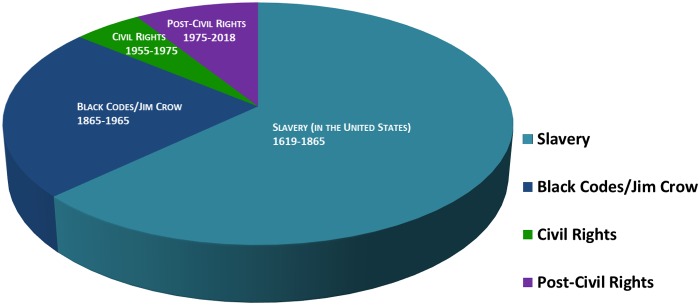
Key periods of Africans and their American descendants in the United States.

Figure 1 presents a time period spanning 399 years (1619–2018) beginning in 1619 when enslaved Africans were brought to the United States and includes slavery, Black Codes/Jim Crow, Civil Rights, and post-Civil Rights.^14^ Table 1 provides a summary of adverse lived personal experiences and health exposures of African American women during four different time periods. We argue that the race-based experiences of these women underlie many of their sexual and reproductive health conditions.

**Table 1.** Historical and Contemporary Sexual- And Reproductive-Related Health and Healthcare Experiences of African American Women

Period	Time span	No. of years	Personal experiences of AAW that contribute to disparities in sexual and reproductive health	Healthcare experiences of AAW that contribute to disparities
Slavery	1619–1865	246	Public, nude physical auction examinations to determine reproductive ability^15,20^; raped for sexual pleasure and economic purpose^19,23^; purposely aborting pregnancies where rape occurred; Jezebel stereotype emerged of black women being hypersexual^115^; generational poverty	Nonconsensual gynecological and reproductive surgeries performed at times repeatedly on female slaves without anesthesia, including cesarean sections and ovariotomy to perfect medical procedures^27,28^
Black Codes/Jim Crow	1865–1965	100	Rape^35^; lynching (genitalia/reproductive mutilation)^36,37,40^; uncertain/unequal civil rights^35^; stereotypes and negative media portrayals continued; generational poverty	Nonconsensual medical experiments continued^27^; poor or no healthcare for impoverished blacks; compulsory sterilization^47^; Jim Crow laws enforced lack of access to quality healthcare services and opportunities; effects of Tuskegee Untreated Syphilis Study on women^49,50^
Civil Rights	1955–1975	20	Lynching, uncertain/unequal civil rights and violence against women to show superiority and control^35^; stereotypes and negative hypersexual media portrayals continued; generational poverty	Nonconsensual medical experiments continued^27,132^; compulsory sterilization^47^; effects of Tuskegee Untreated Syphilis Study on women^50^; unequal healthcare services^30^
Post-Civil Rights	1975–2018	43	Black exploitation movies, media's hypersexual images continued^116–117^; generational poverty	Unequal healthcare continued^30^; targeted sterilizations, hysterectomies, abortions, and birth control^42,43,47,53,54^
Total no. of years	1619–2018	399		

AAW, African American Women.

Race-based mistreatment that occurred during the 246-year enslavement (1619–1865) of Africans and their descendants involved many sexual and reproductive acts of violence against both enslaved African American women and their sexual partners. Enslaved women often experienced legalized sexual and reproductive exploitation.^15–19^ Some sources estimate that 58% of all enslaved women aged 15–30 years were sexually assaulted by slave owners and other white men.^15,20^ Due to laws defining them as property, enslaved women had no legal protection from sexual assault by white men.^19^

Acts of sexual violence against African American men could also affect enslaved women. Because enslaved men were viewed as social threats, and had few criminal justice protections, mobs of white men publicly lynched and/or castrated them in efforts to assert their dominance over them.^21^ In addition to disrupting relationships between enslaved women and their male partners, such occurrences restricted their opportunities to reproduce with a partner of their choosing.

Consequently, childbearing during slavery was often intrinsically related to an economic system that benefitted white slave owners more so than a matter of personal freedom.^15,22^ Because enslaved women and girls were denied reproductive rights to control their own sexuality, they were unable to determine with whom they engaged in sexual relationships.^23,24^ Women who were considered “strong” were sold as breeders and routinely sexually assaulted to birth more children into slavery.^23^ Some enslaved females attempted to avoid being sexually exploited for these purposes and aborted their pregnancies as an act of resistance.^23,25,26^

Enslaved women had limited access to healthcare, and the available “care” often involved medical experimentation.^27^ James Marion Sims, the “Father of Modern Gynecology” and former president of the American Medical Association, performed many reproductive experimental surgeries without anesthesia to treat various childbirth illnesses among enslaved African American women.^28^ Many physicians used enslaved women in other experimental reproductive surgeries, such as cesarean sections and ovariotomy, to perfect procedures that would later be used for all women.^29^

Adverse sexual and reproductive health and healthcare experiences continued for African American women throughout the Black Codes/Jim Crow era. (Table 1).^24,30,31^ Although the Emancipation Proclamation granted freedom to the enslaved, the Black Codes restricted African Americans' labor advancement and migration, and Jim Crow laws restricted their overall civil rights.^32,33^ In some states, laws regarding rape protected only white women although some sources contend that African American women were more often victimized by this crime.^21^ In the absence of laws to protect African American women, rape served to control them, which likely affected their self-esteem and self-worth.^34^ Lynching was also used to punish both women and men who sought racial equality through civil rights.^24,35,36^ Many African American women also endured public gang rape and genital mutilation before being lynched.^37–40^

Furthermore, eugenic programs emerged to control the size of the black population.^41–43^ These programs coerced African American women to undergo sterilizations without their full knowledge that these procedures were not reversible.^44^ Although the eugenic thesis was refuted by scientists, several state-supported eugenic sterilization programs remained active.^45,46^ Thirty states supported formal eugenic programs that enforced compulsory sterilization from the early 1900s to the 1970s.^47^

The longest running medical experiment in the United States was the “Tuskegee Syphilis Study of Untreated Syphilis in the Male Negro.”^48^ Beginning in 1932, the U.S. Public Health Service recruited poor and uneducated African American men in Alabama to determine the effect of untreated syphilis. Although treatment became available, the men were misled, denied treatment, and not informed of the study findings until 1972.^49^ In addition to study subjects experiencing syphilis-related morbidity and mortality, some of their wives acquired syphilis, and some of their children suffered complications from congenital syphilis.^50^

Inhumane healthcare provided during the Black Codes/Jim Crow era was replaced with limited, poor-quality, or no health services for many African Americans, particularly those living in poverty during the Civil Rights era.^30^ Both the Civil Rights and post-Civil Rights eras have been characterized by overt and subtle forms of racism in the U.S. healthcare system. Legal segregation in healthcare continued through the mid-1960s until Congress passed the Civil Rights Act of 1964.^51^ Shortly thereafter, the Medicaid program forced many hospitals to adhere to the Civil Rights Act and to hire doctors who would treat patients of all races, although unequally.^51^ Federal funding supported coerced sterilization, and some African American women were threatened with denial of medical care or termination of welfare benefits if they did not undergo sterilization.^52^ Moreover, in 1972, ∼20 women, mostly young, African American and poor, suffered unintentional abortions as a result of the super coil. The super coil was a device that caused uncontrollable bleeding and, in some cases, led to hysterectomies, abdominal pain, and anemia.^53^

In addition, many poor African American women underwent unnecessary hysterectomies as practice for medical students at select teaching hospitals.^54^ This exploitation of African American women became routine and perpetuated the eugenic movement during this time period.^47^ Although long-acting reversible contraceptives (i.e., implants) are now recommended as the most effective contraception option for many women, including adolescents regardless of race/ethnicity, debates about reproductive justice and the use of these contraceptives among African American women persist.^55,56^ African American women also report experiences of racial discrimination when seeking family planning services, and are more likely than white women to be advised to restrict childbearing, which might engender feelings of mistrust.^57–60^ Likewise, black women of low socioeconomic status (SES) were more likely than white women of low SES to be recommended by their healthcare provider for intrauterine contraception.^61^

Taken together, these historical experiences of sexual violence, experimentation, and healthcare disenfranchisement support the intergenerational transmission of poor sexual and reproductive health outcomes among African American women in the United States.

### Contemporary sexual and reproductive health outcomes

The CDC reports that African American women experience a high burden of STIs, including HIV.^62^ In 2012, compared with white women, African American women were more likely to be diagnosed with primary or secondary syphilis, gonorrhea, or chlamydia (16.3, 13.8, and 6.2 times, respectively).^62^ African American women were also two to three times as likely as white women to have pelvic inflammatory disease.^62^ If left undiagnosed or untreated, these conditions can lead to pregnancy complications and infertility.^62^ In addition, CDC reported that African American women had an HIV incidence rate that was 20.1 times greater than that of white women in 2010.^63^ African American women are also more likely to have delayed HIV treatment compared with women of other races.^64^

Pregnancy-related morbidity and mortality also disproportionately affect African American women.^65,66^ In 2013, CDC reported that the preterm rate for black infants was ∼60% higher than for white infants (17.1% and 10.8% respectively).^67^ In addition, the low birth weight rate for African Americans was 10.13% and 6.97% for whites.^68^ During 1998–2005, African American women had a three to four times higher risk of pregnancy-related death at every age interval compared with women of other races.^69^ African American women also have increased risk for pregnancy-related hypertension and chronic hypertension.^70^ Importantly, this increased risk of mortality suggests that African American women are less likely to receive quality prenatal care and other preventive services (e.g., preconception health counseling and quality care for pre-existing medical conditions such as hypertension).^71^

African American women undergo more hysterectomies due to conditions (e.g., uterine fibroids) that are potentially treatable by less aggressive procedures than other women.^72–74^ Kjerulff et al. also found that black women were more likely than other women to have longer hospital stays and three times the inhospital mortalities, as well as other complications (i.e., respiratory, postoperative infection, gastrointestinal, hemorrhage, hematoma, accidental puncture, or laceration).^74^

Researchers are urged to examine any biases they may have about African American women before interpreting data about their sexual and reproductive health. Although focused on African American men, Leigh and Huff outline important considerations regarding reporting bias that are pertinent for African American women.^75^ First, racism is a social factor embedded within the historical legacy of the United States.^57,75,76^ The effects of racism and unconscious bias are difficult for African American women to avoid, because race and ultimately racism are based on physical characteristics (i.e., skin color). Whether racism is internalized, experienced within institutions (i.e., workplace), or through societal assumptions (i.e., preconceived notions about racial groups), it increases the risks of adverse sexual and reproductive health outcomes for this population.^77^ Second, there may be a reporting bias related to African Americans, because African Americans disproportionately access medical care in publicly funded clinics due to socioeconomic disparities. These clinics typically have more stringent reporting requirements.^75^ Finally, differences in sexual and reproductive health may be exaggerated as African Americans may be more likely to use service providers who use different patterns of testing and reporting.^51^

For example, healthcare systems that emphasize teaching and research related to patient care may have a higher proportion of African American patients, which can lead to the identification of health problems believed to be more common among African Americans (e.g., STIs).^51^ In light of the nuances associated with the collection, analysis, interpretation, and reporting of health data for African American women, some researchers argue that there is an intersection between the health and healthcare experiences of African Americans and the social conditions (e.g., poverty, limited education, residential segregation) they live in, helping shape patterns of documented health inequities, including sexual and reproductive health inequities.^78–81^

### Linking past experiences to current health outcomes

**FIG. 2.**
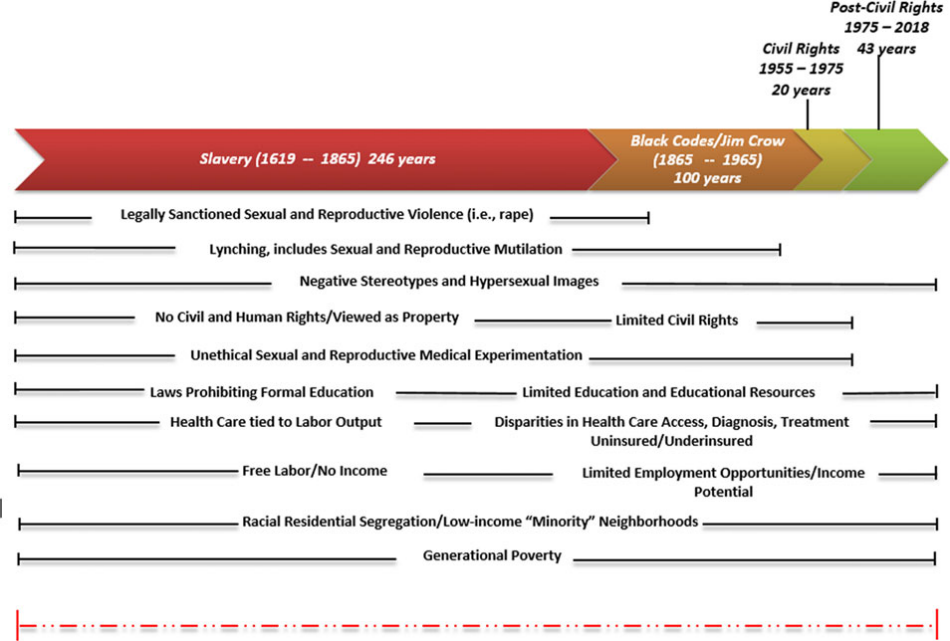
Time line of key historical and contemporary racial and social experiences of Africans and their American descendants in the United States.

The historical context of racism continues to shape the sexual and reproductive health of African American women. Figure 2 is a visual representation of key historical and contemporary social conditions experienced by African American women in the United States. It demonstrates the trajectory of adverse social determinants (i.e., poverty), which may affect the current health status of African American women. Although improvements in the public health and the healthcare system have occurred over time, the following paragraphs discuss the continuum of racism-related experiences that began in slavery and have been found to influence sexual and reproductive health today.

Transgenerational poverty originated in slavery and continues to disproportionately affect African Americans.^82^ Given the well-established link between racism, poverty, and health, the socioeconomic conditions associated with institutionalized and interpersonal racism make African American women more vulnerable to sexual and reproductive health problems.^81^ For example, African American women are more likely than other women to live in neighborhoods in which the HIV prevalence is relatively high,^83^ increasing the likelihood that they will encounter HIV-infected partners.^84^

Limited education may contribute to health issues experienced by African American women. During slavery, laws prohibited enslaved women from receiving a formal education.^14^ In later periods, most African American women had few opportunities for formal education, and black schools were given lower quality educational materials than schools educating white students. Low educational attainment may be associated with multiple sexual and reproductive health issues.^85–88^ Studies show that limited education is associated with an increased likelihood of poor HIV treatment adherence, preterm births and infant mortality, and undergoing hysterectomy.^89–91^ Furthermore, the frequency of hysterectomies among African American women with poor education has amplified concerns about the frequency with which this procedure is used.^91,92^

Race-based residential segregation continues to differentially structure access to quality educational opportunities in many predominantly African American neighborhoods.^93,94^ Ultimately, residential segregation by race provides a foundation to maintain other forms of institutional and societal segregation.^80^ Importantly, it plays a central role in reproductive and sexual health by limiting access to quality health services.^95,96^ For example, African Americans living in predominantly black communities are considerably less likely to receive early HIV testing and treatment than whites.^64,97^ In addition, residential segregation is linked with adverse reproductive health outcomes, which are rooted in social inequalities.^98^

Some researchers have discussed structural inequalities in employment opportunities in relation to sexual and reproductive health outcomes. Historically (i.e., during slavery), African American women were not compensated for the work they performed. As slavery came to an end, they were not provided access to resources or immediate employment opportunities to sustain themselves and their families.^99^ Low-paying jobs with few opportunities for advancement have been found to influence decision-making around sexual behavior. Poverty is associated with sexual risk decisions in efforts to acquire basic needs, such as food and shelter.^100–103^

A personal history of sexual violence may also influence the overall health of African American women.^104,105^ Repeated assaults have been linked to trauma, which can increase the likelihood that women will experience sexual health problems (e.g., sexual dysfunction).^106–108^ African American women living in poverty have an increased likelihood of enduring childhood sexual abuse.^109^ Furthermore, reproductive coercion diminishes self-esteem, resulting in feelings of inferiority, high levels of stress, and vulnerability to sexual risk behaviors.^110^

Present-day stereotypes of African American women as “hypersexual,” “aggressive,” and “angry” were born of representations that emerged in the past.^133,107,111–113^ Negative sexual stereotypes of African American women began as a means to justify their enslavement and subsequent sexual violence, including rape and sexual assault.^114^ Negative sexual imagery of African American women continued throughout the four time periods.^115–117^ Peterson et al. recently found that African American women who reported viewing more sexual stereotypes in rap videos engaged in more sexual risk behaviors than females who did not. Some data suggest that these negative stereotypes help to further racist sentiments because they can be internalized by African Americans.^118^ Men of color who perpetuate these images either intentionally or unintentionally have themselves been victims of persistent negative imagery throughout history that often translates into internalized racism.^119^

Because many African American women lack access to quality healthcare, they have an increased likelihood of late-stage diagnoses of HIV and other medical conditions that increase the risk for early mortality.^30,120^ Many African American women lack access to preventive reproductive screenings, including mammograms and Pap smears.^121^ Some data suggest that factors contributing to disparities in preterm birth risk or infant mortality include differences in prenatal care, nutrition, and SES as well as experiences of racism-related stress.^65,121–125^

Some suggest the origins of adult health begin with intrauterine and early postnatal experiences or as a result of “weathering,” through which repeated experiences with discrimination result in physical health deterioration in early adulthood.^126–129^ Low birth weight among contemporary African Americans has been proposed to be a result of differences in current exposures to social and environmental factors that affect fetal development and from conditions experienced during slavery. Enslaved women endured poor health across their life span due to insufficient diet, extreme physical work, and disease.^130,131^ Jasienska highlights the concept of “fetal programming,” the idea that the physiological development of the fetus can be affected by environmental events, which may endure into adulthood, thereby affecting future generations. Although slavery was abolished in the United States in 1865, Jasienska argues that there has not been enough time to eliminate the physical effects of slavery, which contributes to the disproportionately high levels of low birth weight in African American infants born in the 21st century.^130^ Although there are multiple risk factors for preterm birth and low birth weight, long-term, multigenerational exposure to inadequate nutrition as evidenced during slavery should be considered when addressing low birth rate.^130^

Additionally, the legacy of medical experimentation and inadequate healthcare has exacerbated African American women's complex relationship with healthcare systems, past and present, and laid a foundation of mistrust of the medical establishment.^132,133^ Some researchers argue that the study of African Americans is incomplete if cultural mistrust is not taken into consideration.^134^ Research suggests that African Americans are reluctant to engage in clinical trials and may refuse treatment as a result of their own race-related experiences.^133,135–137^ The lingering effects of the “Tuskegee Study of Untreated Syphilis” on African American women support the need for present-day medical schools to adopt culturally and linguistically appropriate curricula that consider how this study continues to impact the reproductive health and related behaviors of African American women.^50^

### Implications for public health

The historical and contemporary racism-related health and healthcare experiences of African American women to date highlight the need to develop new models for health promotion. Socioecological models are useful for understanding the context of both race-specific and gender-specific issues relative to sexual and reproductive healthcare experiences.^138^ For example, programs designed to address individual-level (i.e., self-esteem, resilience), interpersonal-level (i.e., reducing stigma), community-level (i.e., reducing residential segregation), and importantly system-level factors (i.e., reducing unemployment) might facilitate long-term, sustainable improvements in health for the larger population of African American women.^139^

Consistent with strategies outlined in the Department of Health and Human Services Action Plan to Reduce Racial and Ethnic Health Disparities and Healthy People 2020, we highlight the following strategies as first steps in reversing historical patterns of poor sexual and reproductive health outcomes among African American women: (1) ensure strategies focus on culturally and contextually appropriate research and prevention, (2) ensure equal access to effective sexual health information and quality healthcare services, (3) support quality education and training for public health professionals, and (4) support policies that promote sexual and reproductive health equity.

To ensure strategies incorporate culturally and contextually appropriate research and prevention, an understanding of cultural theories and perspectives is central to prevention efforts. This approach enables the development of programmatic systems and policy actions that are relevant and appropriate for the intended audience. African American women must be involved in the design, implementation, and evaluation of all aspects of the research and implementation of agreed-upon programs. Such an approach is modeled by community-based participatory research.^140^ Similarly, it is important to closely examine macrolevel factors that impact health outcomes, such as the socioeconomic, cultural, and dimensions of the community/environmental context. This approach further illuminates the impact of social determinants of health on African American women and expands opportunities and strategies for primary prevention.

Addressing equal access to effective sexual and reproductive health information and quality healthcare services that stem from institutional racism and discrimination entails reducing barriers to access to quality care, increasing access to health insurance, and ensuring the provision of culturally appropriate and specialized care. The Affordable Care Act (ACA) could improve African American women's access to quality, affordable health coverage and help reduce inequities.^121^ The ACA was designed to expand access for preventive screening services for women, increases maternity coverage, and increases funding to community health centers, which are generally located in disenfranchised communities serving large numbers of African Americans. Moreover, to effectively and efficiently address those underlying causes of adverse sexual and reproductive health outcomes for African American women, public health agencies are encouraged to broaden their partnerships to include nontraditional partners (i.e., housing, education, employment) who might have more direct influence over some of the social determinants affecting the health status of African American women.

Addressing the shortage of African American public health professionals and supporting quality education and training are significant in improving the provision of high-quality healthcare. Their representation in the workforce has both educational service and relationship benefits for patients and providers. In addition, ensuring their presence within the healthcare profession serves as an opportunity to address the discriminatory practices that may have prevented their entry into healthcare professions.^141,142^ Patient/provider relationships are also a factor in achieving patient satisfaction and medication adherence.^143,144^ Effective patient/provider communication is paramount to delivering high-quality health services, and patients are more apt to share information helpful to their provider when they feel valued.^145^

In addition, public health researchers should be familiar with the histories and lived experience of their African American patients to appropriately design collaborative prevention efforts that ameliorate racism and its health-related impacts among African American women. Learning to be culturally competent and sensitive is essential for providers and public health practitioners providing services to populations that have traditionally been marginalized and medically underserved.^145^

Moreover, policies that promote health equity can be powerful tools for social change. Enforcing policies that promote racial and gender equality, quality education for all students, equal access to job training and employment opportunities, and equal access to quality health care for all could enhance population health.^138,146,147^

## Conclusion

The field of public health will be more successful addressing the root causes of health inequities when strategies are informed by rigorous social and epidemiological research. Properly framed and executed, such research can support the development of approaches that take into account the unique experiences of African American women. This overview of historical health-related experiences of African American women is a first step in describing how the historical impact of racism links past events to present sexual and reproductive health outcomes. Addressing sexual and reproductive health through a historical lens and ensuring the implementation of culturally appropriate programs, research, and treatment efforts will likely move public health toward achieving health equity, which will benefit the health of African American women.

## Disclaimer

The findings and conclusions in this report are those of the authors and do not necessarily represent the official position of the Centers for Disease Control and Prevention.

## Notes

### Abbreviations Used

ACA: Affordable Care Act
AAW: African American women
CDC: Centers for Disease Control and Prevention
SES: socioeconomic status
STI: sexually transmitted infections
